# Identification and Functional Analysis of lncRNA by CRISPR/Cas9 During the Cotton Response to Sap-Sucking Insect Infestation

**DOI:** 10.3389/fpls.2022.784511

**Published:** 2022-02-23

**Authors:** Jie Zhang, Jianying Li, Sumbul Saeed, William D. Batchelor, Muna Alariqi, Qingying Meng, Fuhui Zhu, Jiawei Zou, Zhongping Xu, Huan Si, Qiongqiong Wang, Xianlong Zhang, Huaguo Zhu, Shuangxia Jin, Daojun Yuan

**Affiliations:** ^1^National Key Laboratory of Crop Genetic Improvement, Huazhong Agricultural University, Wuhan, China; ^2^College of Plant Science and Technology, Huazhong Agricultural University, Wuhan, China; ^3^Biosystems Engineering, Auburn University, Auburn, AL, United States; ^4^College of Biology and Agricultural Resources, Huanggang Normal University, Huanggang, China

**Keywords:** cotton, CRISPR/Cas9, lncRNA, RNA-Seq, JA signaling, plant-herbivore interaction, sap-sucking insect

## Abstract

Sap-sucking insects cause severe damage to cotton production. Long non-coding RNAs (lncRNAs) play vital regulatory roles in various development processes and stress response, however, the function of lncRNAs during sap-sucking insect infection in cotton is largely unknown. In this study, the transcriptome profiles between resistant (HR) and susceptible (ZS) cotton cultivars under whitefly infestation at different time points (0, 4, 12, 24, and 48 h) were compared. A total of 6,651 lncRNAs transcript and 606 differentially expressed lncRNAs were identified from the RNA-seq data. A co-expression network indicated that lncA07 and lncD09 were potential hub genes that play a regulatory role in cotton defense against aphid infestation. Furthermore, CRISPR/Cas9 knock-out mutant of lncD09 and lncA07 showed a decrease of jasmonic acid (JA) content, which potentially lead to increased susceptibility toward insect infestation. Differentially expressed genes between wild type and lncRNA knock-out plants are enriched in modulating development and resistance to stimulus. Additionally, some candidate genes such as *Ghir_A01G022270, Ghir_D04G014430*, and *Ghir_A01G022270* are involved in the regulation of the JA-mediated signaling pathway. This result provides a novel insight of the lncRNA role in the cotton defense system against pests.

## Introduction

Cotton is an important cash crop that produces natural fiber. As fiber production and quality are impacted by pests, farmers routinely spray broad-spectrum chemical insecticides affecting non-target insects that causes a serious impact on the economy, environment and human health (Lawo et al., 2009; Digilio et al., 2012; Pessoa et al., 2016). Thus, breeding for insect resistance has become a focus in the cotton industry today. By the end of the 1970s, the advent of genetic engineering has revolutionized insect control by developing novel control strategies. The establishment of *Bacillus thuringiensis* (*Bt*) technology for example has greatly reduced the severity of several cotton-attacking insects including cotton bollworm (Zhang W. et al., 2018). However, genetically modified crops lose their efficacy due to the development in pest resistance. Therefore, scientists have switched their interest toward finding more stable, efficient and safer breeding techniques that can overcome previous technologies.

Long non-coding RNA (lncRNA) is a non-coding endogenous RNA regulatory molecule that possesses a mature structure and a length greater than 200 nt (Ulitsky and Bartel, 2013; Jandura and Krause, 2017). According to previous studies, lncRNA plays diverse roles based on its position in the genome (Ma et al., 2013). Generally, lncRNA is involved in important biological processes including affecting chromosome conformation, transcriptional activation and interference, and enzyme activity regulation (Ponting et al., 2009; Derrien et al., 2012; Rinn and Chang, 2012; Heo et al., 2013). In plants, lncRNA are also involved in biotic and abiotic stress responses (Ben Amor et al., 2009; Zhu et al., 2014; Wang J. et al., 2015). Since its discovery, much research has been carried out to understand the mechanisms of lncRNA actions in many mammalian species including mouse and human, with less focus on plants (Sun et al., 2012; Shi et al., 2013). However, in the recent years lncRNAs have begun to attract plant researcher’s attention. In *Arabidopsis*, 6,480 lncRNAs were identified using a bioinformatics approach and obtained 2,708 lincRNAs based on RNA-seq evidence, which provides an important method for the identification of lncRNA in other plants (Liu et al., 2012). In *Populus*, 2,542 lncRNAs were identified and 504 lncRNAs were found to influence drought response (Shuai et al., 2014). A total of 3,170 lncRNAs were identified in rice by RNA-seq analysis under phosphate (Pi) starvation using competing endogenous RNA network to study lncRNAs response to Pi starvation (Xu et al., 2016). In tomato and *Phytophthora infestans* interaction network, lncRNA-16,397 was alleviated by cell membrane injury, resulting in enhanced resistance to *P. infestans* (Cui et al., 2017). A recent study showed lncRNAs involvement in cotton resistance to the pathogen *V. dahlia* (Zhang L. et al., 2018). Additionally, lncRNAs play central roles in insect-resistance interaction. Recent research on cotton has shown that some lncRNA’s were involved in plant response to *Aphid* damage (Zhang et al., 2019).

Although a large number of lncRNAs have been identified in plants, most have unknown function due to their complex structure, low expression level and the absence of sequence conservation. Thus, most published reports have focused on lncRNAs identification rather than their biological function. Recently, the explosion of genome editing technologies has facilitated the study of lncRNA function (Chu et al., 2015). CRISPR/Cas9 is a gene editing technology that has revolutionized the functional genomics studies due to its high efficiency, high stability, simplicity and low off-target effect (Li et al., 2019b; Manghwar et al., 2019, 2020). Recent studies have shown that genome editing of the *Xa13* promoter has improved the rice resistance to bacterial blight disease (Li et al., 2020). In rapeseed, knockout of two *BnaMAXIB* homologous genes through CRISPR/Cas9 has increased plant yield (Zheng et al., 2020). CRISPR/Cas9 has also been used to determine that the loss of function of the *GmPRR37* gene in soybean exhibited early flowering (Wang et al., 2020) while the loss of function of three *BnITPK* gene mutants showed low phytic acid content in rapeseed (Sashidhar et al., 2020). The fitness effect of CRISPR/Cas9 system on lncRNAs genes was also monitored (Horlbeck et al., 2020).

Plant hormones play an indispensable role in plant defense responses (Bari and Jones, 2009). Jasmonic acid (JA) has been found to be an important hormone and its signaling pathway has a direct or indirect defense response to insect damage (Wei et al., 2011; Soler et al., 2013). Previous studies have shown that abscisic acid (ABA) is also an important regulatory hormone that provides insect resistance. Low levels of ABA showed increased susceptibility to herbivore infection (Thaler and Bostock, 2004; Bodenhausen and Reymond, 2007; Dinh et al., 2013). In plants, hormones interact with each other by signaling mechanisms. It has been found that ABA and JA signals collectively induced plant defense against masticatory pests. Studies have also shown that the oral secretion of *Manduca sexta* induced the ABA signaling pathway, whereas, enhancing the JA-mediated signal defense response was under consideration (Dinh et al., 2013). Recent research showed that overexpression of *GhLac1* in cotton *Gossypium hirsutum* leads to the rapid accumulation of Jasmonic Acid (JA) and secondary metabolites, resulting in increased resistance against *Verticillium dahliae* and cotton bollworm (Hu et al., 2018).

With the completion of cotton genome sequencing data (Li et al., 2015), it is feasible to identify lncRNA throughout the genome. Therefore, it is important to explore the function of lncRNA in cotton associated with insect resistance. The overall objective of this work was to determine whether lncRNAs are resistant to sucking insect pests. The specific objectives were (1) Identify novel lncRNAs from the RNA-Seq dataset of cotton tissue samples infested by whitefly *Bemisia tabaci*. (2) Generation of targeted mutation by CRISPR/Cas9 to explore the role of lncRNA in cotton. (3) RNA-Seq analysis of CRISPR/Cas9 knock-out cotton plants and endogenous determination of plant hormone between transgenic and control lines. This study reveals that lncRNAs play a critical role in plant response to insects.

## Materials and Methods

### Experimental Materials

Whitefly resistant (HR) and susceptible (ZS) cotton seeds were sown in 1/2 Murashige and Skoog (MS) medium. After germination, seeds were placed in the dark for 2 days and then put in a growth chamber at 26°C with a 16 h/8 h day light/dark cycle until they grew cotyledons (Li et al., 2019a). Fifty adult whiteflies were placed on the cotton cotyledons. The leaves of HR and ZS cotton cultivars infested by whitefly aphids were collected, respectively, at different time points (0, 12, 24, and 48 h) with three replicates. Total RNA was extracted using a modified guanidine thiocyanate method from each leaf collected at each time interval for each replication (Tu et al., 2007).

### Identification of lncRNA

The identification of lncRNA process monitored as following: (1) 22 forms of RNA-Seq (SRA project: PRJNA286935) were aligned to *G. hirsutum* acc. TM-1 genome (Trapnell et al., 2012; Zhang et al., 2015) using TopHat 2.0 (Trapnell et al., 2012; Zhang et al., 2015) with default parameter. (2) The cufflinks 2.0 (Trapnell et al., 2010) program was used to assemble the transcript and calculate the gene expression. Cuffmerge was then used to merge the gtf files, and then the gtf files were reassembled and transcripts were compared by cuffcompare. There were several types of class codes for the newly assembled transcripts. Based on the location of the lncRNA on the mRNA, class “x” was assigned for antisense transcript, “i” for intron and “u” for intergenic region (Trapnell et al., 2012). (3) We screened the lengths less than 200 bp with an FPKM value > 0.5 for at least one sample. (4) For the evaluation of protein coding ability, the identified lncRNAs were aligned to the Pfam and SwissPort database using the blast program, and the observed lncRNAs that were not aligned were kept for further evaluation by the CPC and CNCI software (Sun et al., 2013). (5) Excluding the putative lncRNAs which were located 500 bp upstream or downstream gene region. (6) LncRNA sequences have supported by expressed sequence tags (ESTs) data and 454 sequencing datasets (Wang M. et al., 2015).

### Functional Analysis of lncRNA

The functional analysis of lncRNA was based on two methods: (1) The hub gene method, which is the expression of lncRNA and differentially expressed mRNAs used to construct the co-expression network using Cytoscape (Shannon et al., 2003). (2) Cluster analysis of lncRNA and mRNA based on Gene Ontology (GO) enrichment analysis to speculate the potential function of lncRNA.

### Construction of Weighted Gene Co-expression Network

For detection of gene co-expression modules, co-expression network analysis was performed on lncRNA and different expressed genes (DEGs) expression profiles using an R package WGCNA (Langfelder and Horvath, 2008). WGCNA builds a network in which genes are treated as points, and relationships between genes are treated as lines (Zhang and Horvath, 2005). The correlation between genes were calculated based on gene expression values and then weighted to make the whole network approximate the scale-free network distribution, including soft threshold selection, adjacency matrix calculation. Finally, the co-expression network is visualized using the Cytoscape software (Shannon et al., 2003).

### CRISPR/Cas9 Vector Construction

For vector construction, sgRNAs were designed according to CRISPR-P web tool (Liu et al., 2017). For lncD09 and lncA07 gene editing, two sgRNAs were designed for each, respectively, namely sgRNA1 and sgRNA2. The CRISPR/Cas9 vector used in this report is modified from *pRGEB32-GhU6.9* according to our report (Wang et al., 2018). These two sgRNAs were integrated in a single vector, which included the fragments containing tRNA-sgRNA1 and tRNA-sgRNA2 fusion using pGTR as template, namely PTG (Xie et al., 2015), and then these two fragments were fused together with an overlapping extension PCR. The PTG fragment was ligated to *pRGEB32-GhU6.9-NPT II* expression vector, which was transformed into *Agrobacterium* tumefaciens strain for stable cotton transformation. The primers used in vector construction are listed in Supplementary Tables 1, 2.

### Agrobacterium-Mediated Genetic Transformation of Cotton

The CRISPR/Cas9 vector was introduced into *Agrobacterium* strain GV3101 by electroporation. *G. hirsutum* acc. JIN668 was used as the transformation receptor (Li et al., 2019b). After the seeds were sterilized, the sterilized seeds were placed on a dark culture at 28°C for 4–6 days, and the hypocotyls were cut into 5∼10 mm lengths, and then followed our previously reported method for genetic transformation (Sun et al., 2018; Wang et al., 2018).

### On-Target Mutation Analysis by Hi-Tom

Genomic DNA was extracted from T0 transgenic (refers to section “CRISPR/Cas9 Vector Construction”) and wild type (WT) cotton plants using a DNA quick Plant System (TIANGEN Biotech, Beijing, China). Specific primers were designed before and after the targeted position to amplify the target sites. The Hi-Tom method was then used to analyze the editing efficiency of the target (Liu et al., 2019). The primers are listed in Supplementary Table 3.

### Quantitative Real-Time PCR Analysis

For qRT-PCR, 3 (μg RNA was reverse-transcribed using M-MLV (Promega). An ABI 7500 real-time PCR system was used to perform the qRT-PCR. Relative gene expression levels were calculated using the 2–ΔCt method (Min et al., 2014). To standardize RNA content, expression levels were normalized to *GhUBIQUITIN7* as an internal control.

### Insect Bioassay

In the previous experiment, we obtained the knock-out positive plants in the T0 generation using the Hi-TOM, which is a platform for high-throughput tracking mutations induced by CRISPR/Cas9 (Liu et al., 2019). Then seeds obtained from T0 positive plants were peeled and sterilized with mercury, and then sown in a sterile seedling medium containing Kanamycin+ antibiotic. After germination, seeds were cultivated in the dark at 28°C for 1 day. The seedlings were then placed in the light culture room to continue the cultivation for 4–5 days. They were then transferred to a nutrient rich bowl for hydroponic cultivation in the light culture room for 5–6 days. Finally, grown plants were moved to the cotton greenhouse, and given suitable growing conditions for 50 days. The two cotton plants were seeded in the same nutrient bowl. One plant was considered as the transgenic line, and the other one was the JIN668 control line. After 1 week, damage by aphids was observed and the number of aphids on each plant was counted.

### Determination of Hormone Content

We use the third true leaf of the T1 cotton plants whose lncRNA was successfully knocked out for the determination of hormone content. 0.05 g of leave sample was collected and kept in 2 ml tubes, grinded in pestle mortar by the help of iron balls and put on shaking using 80% methanol and placed them all for overnight at 4°C. Plant hormone measurements were performed on an Agilent 4000Q-TRAR HPLC-MS system (Applied Biosystems) (±) −9, 10-Dihydrojasmonic acid and 2H6-ABA used as the internal standard for JA and ABA (Ma et al., 2018).

### Statistical Analysis

The Student’s *t* test was used to compare the means between two groups, whereas ANOVA was used to compare the means among three or more groups and the least significant difference (LSD) method was used to perform multiple comparison (Mishra et al., 2019).

### Transcriptome Analysis of the Genome Edited Cotton Plants by RNA-Seq

The third true leaf of the T1 cotton plants whose lncRNA was successfully knocked out for RNA extraction, two biological replicates and two technical replicates were used (totally four replicates). In total, 10 libraries were constructed, including one control library and four gene-knockout libraries for each lncRNA gene. The filtered clean reads were first matched to the *G. hirsutum* genome (Wang M. et al., 2019) using TopHat 2.0 (Trapnell et al., 2012). The alignment files were used to assembled the transcripts by Cufflinks (Trapnell et al., 2010), and the expression level of the transcripts were calculated. The differential genes expression through screening criteria were elaborated: | log2 (fold change)| > 1 and *P*-value < 0.05 of the gene expression which showed abundance in ratio between samples. Differentially expressed genes were clustered with Cluster Profiler (Yu et al., 2012). The metabolic pathway for gene enrichment was analyzed through functional cluster and KOBAS (Xie et al., 2011).

## Results

### Genome-Wide Identification of Long Non-coding RNA

**FIGURE 1:**
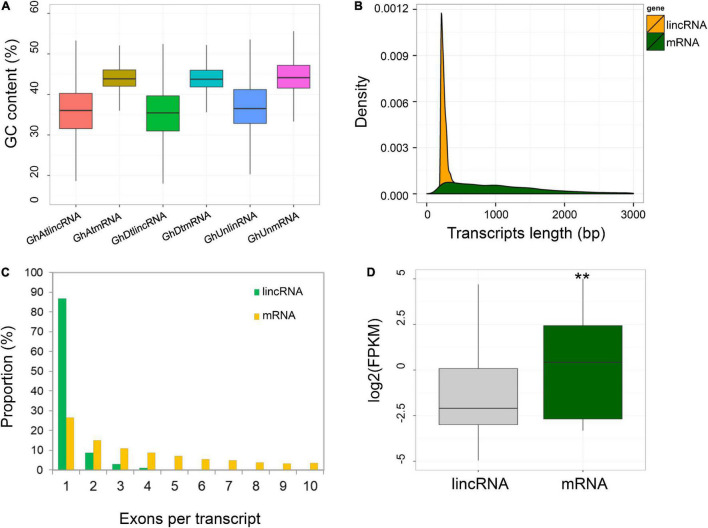
Identification and characterization of cotton lncRNAs expressed in response to whitefly infestation. **(A)** The GC content of lincRNA and mRNA transcripts in the At- and Dt-sub-genome and ungrouped *G. hirsutum* genome scaffolds (Wilcoxon rank sum test, *P* < 2.2e-16). **(B)** Comparison of lncRNA and mRNA transcript lengths. **(C)** The number of exons in the lincRNAs and mRNAs. **(D)** Comparison of transcript expression levels (FPKM) between lincRNAs and mRNAs. **Represents *p* < 0.01.

In this study, 22 RNA-Seq datasets were used to identify the long non-coding RNAs related to cotton-resistance against *B. tabaci* (Li et al., 2016). Since the RNA-Seq library was non-chain-specific, only the lncRNAs present in the intergenic region were selected for further analysis. A total of 6,651 strand-specific lncRNA genes were identified. Although the presence of GC content in lncRNAs was lower than that of mRNAs (median 36.0 vs. 43.6%), there was no significant difference between the At- and Dt-subgenome in the GC content of lncRNAs (Figure 1A). Furthermore, the average transcript length of the respective lncRNAs was shorter than mRNAs (357 vs. 1,180 bp) (Figure 1B). Subsequently, most of the lncRNAs comprised fewer exons (>90% consist of 1–2 exons) than mRNAs (Figure 1C). Next, the expression level of each transcript was estimated based on the median FPKM values, which indicated that lncRNAs were expressed at lower significant level than mRNAs ranging from (0.24 vs. 2.04) (Figure 1D).

**FIGURE 2:**
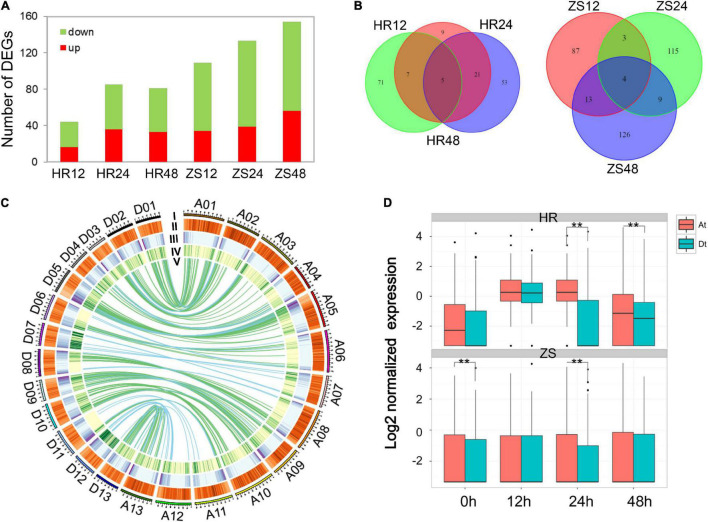
Differentially expressed lncRNAs in cotton response to whitefly infestation. **(A)** Analysis of differentially expressed lincRNAs between HR and ZS plants at different time points during whitefly infestation. **(B)** The number of lincRNAs in different groups. **(C)** Identification of lincRNA and pre-miRNA pairs in the At- and Dt-sub-genome of *G. hirsutum*. I: Chromosome length; II–IV: Distribution transposable element, mRNA, and lincRNA densities in 1 Mb size windows; V: Alignment of lincRNAs and pre-miRNAs between the At- and Dt- sub-genome. Purple and green links indicate the lincRNA and pre-miRNA pairs, respectively. **(D)** Expression profile of homologous lincRNAs in the At- and Dt-sub-genome of cotton following whitefly infestation (Wilcoxon rank sum test, **represents *p* < 0.01).

To systematically evaluate the expression pattern of lncRNAs and their response to whitefly infestation in both HR and ZS plants, the analysis of differentially expressed lncRNAs (DEL) were performed by DESeq (log2 | infestation/control| > 2 and *P* < 0.05). A total of 606 lncRNAs were differentially expressed in the HR and ZS plants after whitefly infestation, and the majority of DELs showed down-regulated behavior (Figure 2A). Interestingly, we also noted that a higher ratio of DELs was found among the lncRNAs (25.6%) than that of mRNAs (3,720 DEGs, 5.3%). There were fewer overlapped lncRNAs than the differentially expressed ones at the different time points in each cultivar under whitefly infestation, and more lncRNAs were differentially expressed in each treatment (Figure 2B). Using the reciprocal align features of BLASTN filtered with MCScanX (Wang et al., 2012), 219 homologous lncRNA pairs were identified between the At- and Dt-subgenome that were located in transposon element (TE) regions (indicated by green color in Figure 2C). We next investigated the expression profile of lncRNA pairs in At- and Dt-subgenome of the HR and ZS plants after whitefly infestation (Figure 2D). Most of the lncRNA pairs were constantly down-regulated in the Dt-subgenome rather than the At-subgenome of HR plants, suggesting that these lncRNAs were specifically repressed in response to whitefly infestation.

**FIGURE 3:**
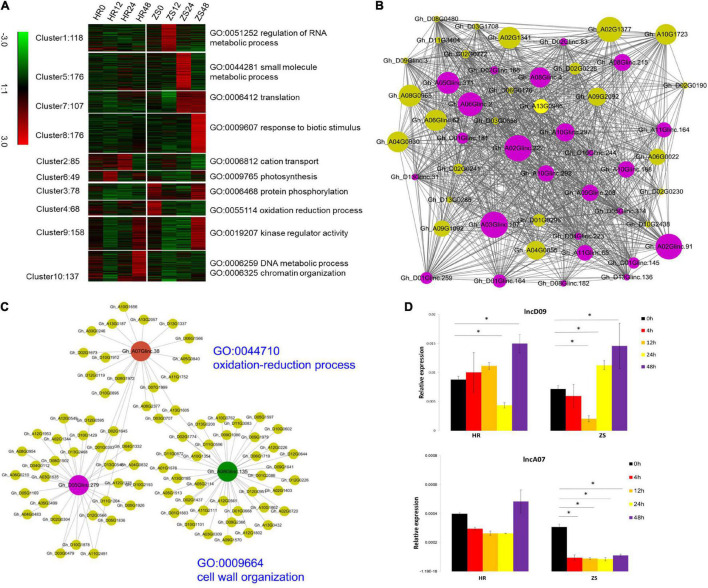
The prediction of lncRNA function and verification of the spatio-temporal expression profile of the selected lncRNAs between the HR and ZS plants after whitefly infestation. **(A)** GO enrichment analysis of lincRNAs (Fisher’s exact test, *P* < 0.05). **(B)** A co-expression network is shown. Each node represents one gene, while each line connects two nodes. The purple and green nodes represent lincRNAs and protein-coding genes (PCGs), respectively. The size of the nodes represents the gene interactions. **(C)** Three lincRNAs interacted closely with PCGs. **(D)** qRT-PCR analysis of lncD09 and lncA07 expression in cotton following whitefly infestation. The *y*-axis indicates the relative expression level (REL), which was calculated based on the 2^–ΔΔCt^ ratio using *GhUBQ7* as a reference. Error bars indicated the standard deviation (S.D) across two biological replicates. *Represents *p* < 0.05.

The potential function of these lncRNAs were analyzed using the *K-means* (*k* = 10) method, 1,178 lncRNAs were abundantly expressed (at least one sample FPKM > 0.5), while 3,720 were differentially expressed protein-coding genes (PCGs). These transcripts were grouped into clusters based on the enrichment of “biological process” GO annotation (*P* < 0.01). The 176 lncRNA transcripts comprising the cluster 8 corresponded to the biotic stimulus response, which is the most efficient enriched GO group in the lncRNA expression profile (Figure 3A). We also created a co-expression network by using a soft threshold power (β = 8; cut-off = 0.9) with higher adjacency (Supplementary Figure 1). The co-expression network contained 50 genes and 1,008 gene interactions, of which the PCGs were the most probable protein kinases observed (Figure 3B). To make the construction of the co-expression network more meaningful, the specific networks were constructed using 28 lncRNAs to produce miRNA precursors and 3,720 DEGs. In this network, the only three hub lncRNAs (*Gh_A07Glinc.38, Gh_A08Glinc.135*, and *Gh_D05Glinc.279*) were directly connected with 20, 38, and 30 PCGs, respectively (Figure 3C). These PCGs were involved in the oxidation-reduction process and cell wall organization (*P* < 0.05). Two lncRNAs (lncD09 and lncA07) from cluster 8 were selected for initial RT-PCR which was based on their spatio-temporal expression profile in HR and ZS plants at the respective five time points (0, 4, 12, 24, and 48 h) during whitefly infestation (Figure 3D). Additionally, the expression of lncD09 was reversed in the HR and ZS plants followed by whitefly infestation at the later time points (Figure 3D). Whitefly infestation for the ZS plants promoted a clear reduction in lncA07 expression that was observed throughout the infestation, whereas in HR plants, infestation had no significant difference in gene expression as compared to 0 h which remained stable throughout the tested time points. These results indicated that the expression of lncRNA was dynamic and sub-genomic divergence occurred under the infestation of *B. tabaci* infection at different time points between two cotton cultivars.

### Detection of Two lncRNAs Coding Protein Ability and lncRNA Expression Analysis

**FIGURE 4:**
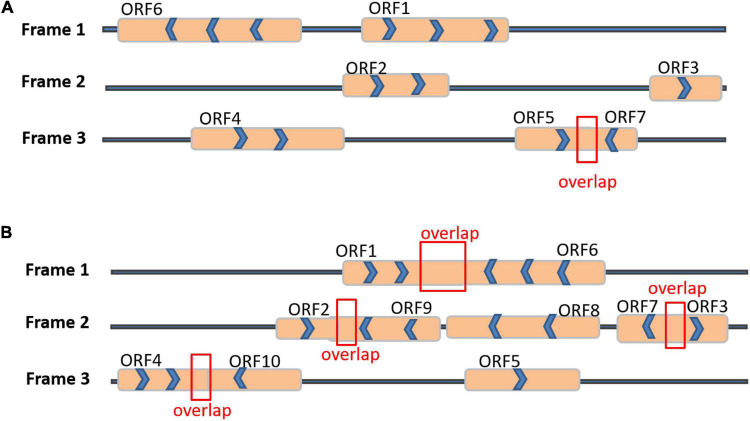
LncRNA ORF prediction and relative expression in different tissues. **(A)** Seven coding short peptide sequences were predicted by lncD09 gene. **(B)** Ten coding short peptide sequences were predicted by lncA07 gene.

Two differentially expressed lncRNA genes obtained from the interaction between cotton and *B. tabaci*, namely lncD09 (sequence length 1,930 bp) and lncA07 (sequence length 727 bp), were identified from the transcriptome data. Both genes differentially expressed at different time points exhibiting resistance to cotton materials (Figure 3D). Firstly, the coding ability of these two lncRNA genes was evaluated by predicting the three open reading frames through query tools. The lncD09 gene contained seven short peptide sequences (Figure 4A), the longest coding short peptide sequence included 47 amino acids. A search alignment was performed to the relevant proteins using the SMART database (Letunic et al., 2012). However, the relevant proteins were not matched in the SMART database. Then, we tried to align the short peptide sequences using Pfam database (Finn et al., 2016), which also couldn’t detect any matched proteins. So, these results indicate that the lncD09 does not have coding capabilities. The lncA07 gene consisted 10 coding short peptide sequences in the ORF Finder (Figure 4B), whereas, the longest coding short peptide sequence contained 71 amino acids. Sequence alignment of lncA07 protein failed to match any homologous sequence by either SMART or Pfam databases which demonstrates that the lncA07 has no ability to encode protein too.

### Generation of Targeted Mutation in the lncD09 and lncA07 Genes by CRISPR/Cas9

Knockout vectors for lncD09 (sequence length 1,930 bp) and lncA07 (sequence length 727 bp) were constructed by using the CRISPR/Cas9 web tool targeting two locus for each gene. The target sequences were inserted into the pRGEB32 (K+) vector by two rounds of PCR. The results were checked through the Gel Documentation system (Supplementary Figure 2). The cotton hypocotyls of JIN668 cultivar were infested with *Agrobacterium* transformation method, which went through subsequent steps of tissue culture to obtain transgenic plants (Supplementary Figure 3).

**FIGURE 5:**
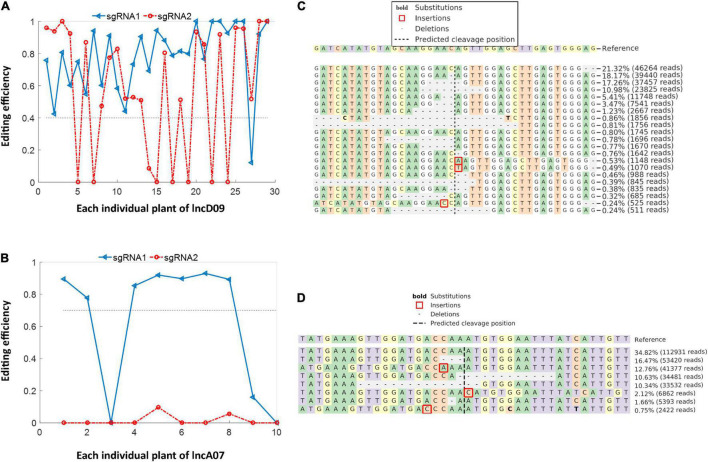
Editing efficiency and editing type of knock out mutants. **(A)** Analysis of editing efficiency of lncD09 in T0 generation. **(B)** Analysis of editing efficiency of lncA07 in T0 generation. **(C)** Analysis editing type of lncD09. **(D)** Analysis editing type of lncA07.

The total genomic DNA was extracted from of the independent obtained transgenic lines to detect the positive plants, results showed that 29 and 10 T0 positive plants were obtained from lncD09 and lncA07, respectively. The obtained positive plants were subjected to subsequent deep-targeting sequencing to detect the editing efficiency. The result showed that the obtained lncD09 plants exhibited different editing efficiency ranging between 40 and 100% (Figure 5A). Among the 10 T0 plants of lncA07, the editing efficiency of sgRNA1 for seven individual plant was more than 70% at the sgRNA1, whereas editing efficiency of sgRNA2 seemed to be extremely low or nearly negligible (Figure 5B). The editing site of both genes was consistent with the predicted cleavage site (3 bp before the PAM). The editing types detected in lncD09 plants were deletion, insertion and substitution in which deletion was the main editing type with deletion length about 1∼20 bp (Figure 5C). The site was consistent with the predicted cleavage site (3 bp before the PAM). The editing of lncA07 mainly exhibited similar deletion pattern to that of lncD09 (Figure 5D).

### Knock-Out Mutants Had a Negative Effect on Aphid Resistance

**FIGURE 6:**
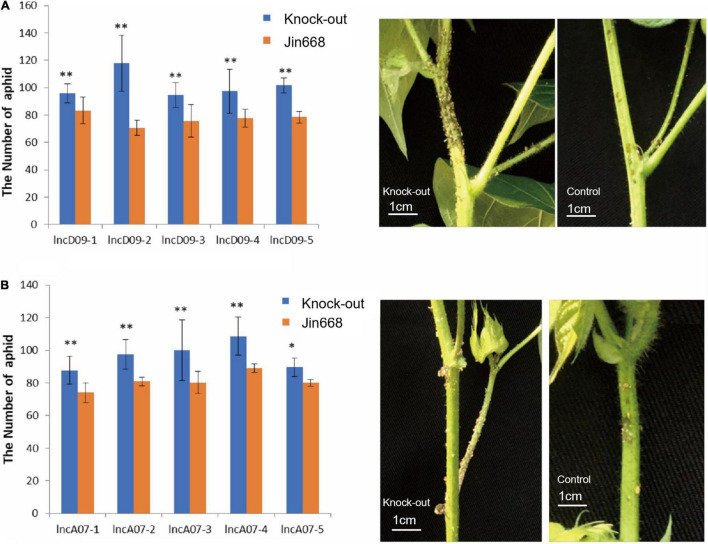
Study on insect resistance of T1 transgenic lines. **(A)** Statistics on the results of aphids in each line of lncD09 gene and picture of aphid infestations. **(B)** Statistics on the results of aphids in each line of lncA07 gene and picture of aphid infestations; Error bars represent the standard deviation of three biological replicates; ^**^Represents *p* < 0.01, *represents 0.01 < *p* < 0.05.

Based on transcriptomic data, lncD09 and lncA07 are lncRNA responsive genes involved in biotic stress response. We first tested the response of the transgenic lines to aphids infestation, plants of JIN688 cultivar were used as control. The genetically modified lines as well as the control were planted at the same time. When the aphids were close to the growing point at the stem tip, the number of aphids were counted at the upper area from the third segment after 50 days. The statistical analysis results showed that the number of aphids in the transgenic lncD09 plants was significantly higher than in the control (Figure 6A). There were some differences observed in the number of aphids between the lncA07 transgenic lines and the control (Figure 6B). However, the insect susceptibility was weaker to lncA07 than lncD09 plants. It appears that knocking out lncD09 and lncA07 genes may cause a decreased resistance against herbivorous sucking insect.

### Transcriptome Analysis of CRISPR/Cas9 Knock-Out Cotton Plants

**FIGURE 7:**
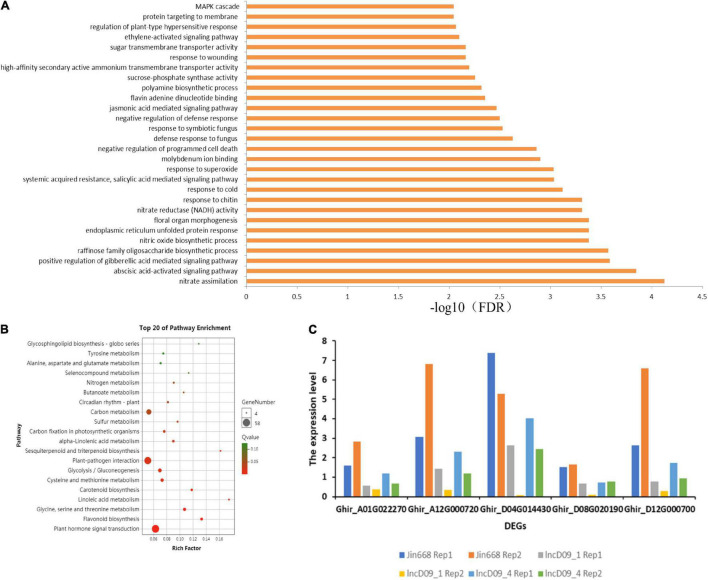
Transcriptome data analysis of knock-out mutants. **(A)** Gene ontology (GO) enrichment analysis of lncD09 mutants. **(B)** KEGG pathway annotation information and analysis of the first 20 enrichment pathways of differentially expressed genes. **(C)** The expression level of DEGs in JA mediated signaling pathway.

To further study the regulatory function of genes related to lncD09 and lncA07, we performed RNA-seq analysis to compare the expression pattern between the transgenic and wild-type (WT) plants in four replicates. The 204 differentially expressed genes [| Log2 (fold change)| > 1, *P*-value < 0.05] were identified in comparison to the lncD09 and JIN668 group (Supplementary Table 4). The GO enrichment analysis showed that the differentially expressed genes of lncD09 were significantly enriched in the ABA-activated signaling pathway, nitrate assimilation, JA-mediated pathway and MAPK signaling pathway (FDR < 0.0001) (Figure 7A). Differentially expressed genes of lncD09 were compared with the KEGG database to screen out enriched metabolic pathways. The result showed that the main enrichment pathways were carbon metabolism, plant-pathogen interaction, plant hormone signal transduction, flavonoid biosynthesis, glycolysis and gluconeogenesis, cysteine and methionine metabolism (Figure 7B). These metabolic pathways play an important role in plant development and resistance to stimuli. We also manually searched all DEGs, and found that the respective genes involved in the JA-mediated signaling pathway were *Ghir_A01G022270*, *Ghir_D04G014430*, and *Ghir_A01G022270*. We found that the expression level of all these genes was significantly inhibited in the knock-out mutants (Figure 7C). This result indicates that the lncD09 is associated with resistance to stimuli, however, the JA-mediated signaling pathway provides strong evidence to study the potential function of lncD09.

**FIGURE 8:**
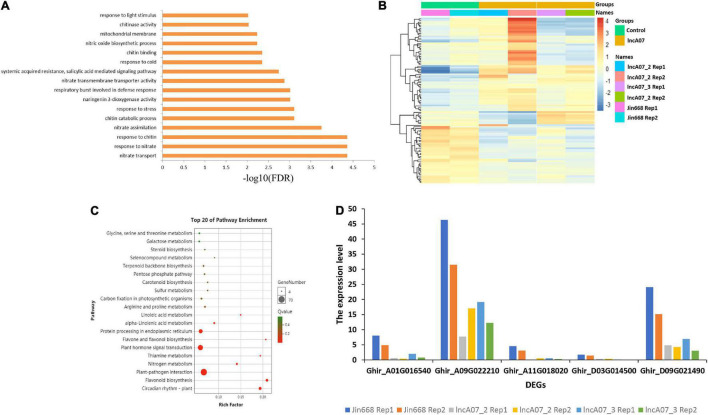
Transcriptome data analysis of knock-out mutants. **(A)** Gene ontology (GO) enrichment analysis of lncA07 mutants. **(B)** The control and transgenic materials demonstrated different gene expression patterns. **(C)** KEGG pathway annotation information and analysis of the first 20 enrichment pathways of differentially expressed genes. **(D)** The expression level of DEGs in chitinase activity.

Additionally, a heatmap was created that showing the relative expression of differentially expressed genes in lncA07. The control line and transgenic lines demonstrated the different gene expression patterns (Figure 8B). Total of 1,186 differentially expressed genes [| Log2 (fold change)| > 1, *P*-value < 0.05] were identified in lncA07 compared JIN668 groups (Supplementary Table 5), this result indicates a wider range of lncA07 regulation. Furthermore, lncA07 differentially expressed genes were classified using GO analysis, and results showed that nitrate transport, response to nitrate and chitin pathways were mainly enriched (FDR < 0.0001) (Figure 8A). Differentially expressed genes of lncA07 were compared by the KEGG database to screen out the enriched metabolic pathways. The results showed that the differential expressed genes were generally enriched in the environmental adaptive metabolic pathway. The top 20 enriched pathways were mostly involved in circadian rhythm-plant, plant-pathogen interaction, plant hormone signal transduction, protein processing in endoplasmic reticulum, flavonoid biosynthesis and the pentose phosphate pathway related to the biosynthesis of steroid skeletons, which are important substances that enhance cotton resistance. In addition, these results were also enriched with a number of pathways related to basic metabolism such as, glycine, serine and threonine metabolism, linoleic acid metabolism, galactose metabolism and nitrogen metabolism (Figure 8C). These metabolic pathways play an important role in cotton response to biotic and abiotic stresses. We screened the genes involved in chitinase activity and found that their expression levels were significantly inhibited in the knock-out mutants (Figure 8D). Because chitinase plays an important role in plant defense response (Nagpure and Bharti Gupta, 2013), the loss of function of lncA07 may also affect the plant resistance to insects.

### Jasmonic Acid and Abscisic Acid Content Are Reduced in lncD09 Mutant Plants

**FIGURE 9:**
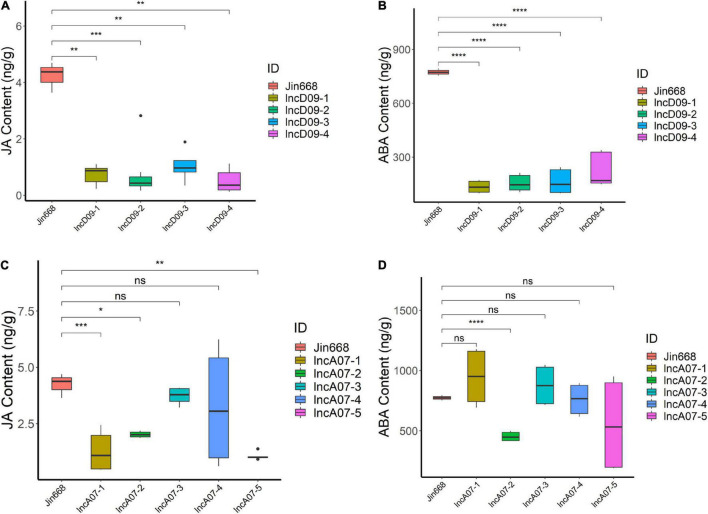
The change of hormone content in transgenic plants. **(A)** The JA content in different lncD09 lines and the control line. **(B)** The ABA content in different lncD09 lines and the control line. **(C)** The JA content in different lncA07 lines and the control line. **(D)** The ABA content in different lncA07 lines and the control line. *Represents *p* < 0.05, **represents *p* < 0.01, ***represents *p* < 0.001, ****represents *p* < 0.0001, ns represents *p* > 0.05.

Many previous studies reported that the JA level was decreased during insect attack (Bodenhausen and Reymond, 2007). Combining the result of the insect resistance trial and transcriptomic analysis, we speculated that it might be caused by hormonal change in knock-out mutants. In order to correlate the endogenous level of JA with the JA-mediated signaling pathway, we performed endogenous determination of hormones for the transgenic and control lines. The result showed that there was less JA accumulated in lncD09 mutants compared to the control plants (Figure 9A). Previous studies have found that ABA and JA signals jointly induced plant defense against insects (Dinh et al., 2013). For further confirmation, we checked the ABA contents in all mutant lines and found that it also showed a decrease in the lncD09 plants compared with control (Figure 9B). On the other hand, JA content exhibited different levels in the different lncA07 lines, in which three out of five lines exhibited significant reduction in JA content (Figure 9C), while ABA level differed significantly in only one line (Figure 9D). These difference might be due to the different editing efficiency among the different lines. Therefore, all these datasets indicated that the overall level of JA were downregulated in lncD09 and lncA07 plants, which increased the susceptibility of mutant plants to insect attack. Although the mechanism of lncD09 and lncA07 mediated JA-signaling is unclear, these results indicated the important role of lncD09 and lncA07 in plant response to pests.

## Discussion

As a novel transcript, lncRNA length is greater than 200 nt and rarely displays the protein encoding sequences. However, the specific regulatory mechanism of lncRNA is not yet completely known, so it is no longer to be considered as “transcription noise.” LncRNA is transcribed in the same transcription mechanism as miRNA containing a 5′- head and 3′-tail. Therefore, lncRNAs can be identified by taking the advantage of high-throughput RNA sequencing technologies that facilitated the identification of lncRNAs in plants (Zhang et al., 2014). So far, the relative studies are still stalled for the identification of lncRNAs from hundreds of RNA-seq datasets, with few studies focused on their regulatory functions. From our present findings, lncRNAs were identified from the transcriptomic data. These lncRNAs showed lower expression levels than protein-coding genes in cotton, and lower GC content than mRNA. In addition, according to the previous reported data, lncRNA has the potential to encoded short peptides, therefore, we calculated the efficiency of lncD09 and lncA07 to encode short peptides. Based on the calculated results and transcriptome data analysis, we speculated that the two genes are genuine for the analysis of long non-coding RNA.

CRISPR/Cas9 is a genome editing technology that modifies a DNA specific sequence, resulting in loss of function in many genes. Therefore, it is an ideal and effective system to knock out desired lncRNA genes that can overcome the limitations of low expression profile. In this study, we constructed CRISPR/Cas9 knockout vectors of the two lncRNAs genes, and successfully obtained positive transgenic plants through the *Agrobacterium*-mediated genetic transformation method. The generation and identification of these materials have provided a chance for further functional analysis of lncRNA.

In this study, we performed RNA-seq analysis to compare the expression pattern between the transgenic and wild-type (WT) plants. The KEGG and GO results show that lncD09 participated in the process of carbon metabolism, plant-pathogen interaction, plant hormone signal transduction. We found that the respective genes involved in the JA-mediated signaling pathway were *Ghir_A01G022270*, *Ghir_D04G014430*, and *Ghir_A01G022270*. Calculating their expression levels by FPKM values, the results showed that the expression levels of these genes were significantly suppressed in the knock-out mutants. Additionally, the RNA-seq result of lncA07 shows that differentially expressed genes enriched pathways were involved in plant-pathogen interaction, plant hormone signal transduction, protein processing in endoplasmic reticulum, flavonoid biosynthesis and the pentose phosphate pathway related to the biosynthesis of steroid skeletons, which are important substances that enhance cotton resistance. According to previously reported work, plant development and stress adaptation rely on the complex network of synergistic and antagonistic mechanisms among various hormones (Howe and Jander, 2008; Schmelz et al., 2009; Erb et al., 2012). JA plays an important role in abiotic stresses, especially in plant resistance to pests. Some studies have confirmed the co-regulation patterns between ABA and JA caused by biotic stress (Dinh et al., 2013; Li et al., 2019a; Si et al., 2020). The knock-out mutants of the lncD09 showed decrease in JA and ABA content. JA content exhibited different levels in the different lncA07 lines, in which three out of five lines exhibited significant reduction in JA content. This reduction was coupled with reduced insect resistance and these results indicate that lncD09 and lncA07 may be a positive regulator in cotton resistance against sap sucking insects. However, the process of lncRNA participated in plant hormone signal transduction and the molecular regulation mechanism are still unclear, it provides a possible road to explore the function of lncRNA in plant defense.

## Conclusion

In this study, transcripts of the JIN668 control and lncD09 gene-edited transgenic lines were compared. Enrichment analysis of GO and KEGG differentially expressed genes showed that lncD09 and lncA07 gene tended to participate in the environmental adaptation process as well as in the plant hormone signal transduction pathway. LncD09 is also involved in the process of energy production and utilization, such as redox reactions, glycolytic reactions and other processes. These results indicated that the lncD09 gene plays an important role in responding to biotic and abiotic resistance in cotton. The results of GO and KEGG enrichment analysis showed that lncA07 plays an important role when cotton was exposed to an external stimulus. It was also related to cell wall modulation, external packaging structure, and chitinase activity. Chitinase was involved in cell wall modification and plays an important role in regulating the growth and development of cotton and responding to stress. RNA-Seq results further confirmed that these two lncRNAs play an important role in insect resistance in cotton.

## Data Availability Statement

The datasets presented in this study can be found in online repositories. The names of the repository/repositories and accession number(s) can be found below: NCBI SRA; PRJNA769752.

## Author Contributions

SJ contributed to the conception and design of the study. SJ, DY, and MA critically revised the manuscript. JZh wrote the manuscript and conceived and designed the study. JL analyzed the data and drafted the manuscript of data analysis. WDB, SS, QM, FZ, JZo, ZX, HS, and QW participated in the study and data collection. All authors have read and approved the manuscript.

## Conflict of Interest

The authors declare that the research was conducted in the absence of any commercial or financial relationships that could be construed as a potential conflict of interest.

## Publisher’s Note

All claims expressed in this article are solely those of the authors and do not necessarily represent those of their affiliated organizations, or those of the publisher, the editors and the reviewers. Any product that may be evaluated in this article, or claim that may be made by its manufacturer, is not guaranteed or endorsed by the publisher.

## Supplementary Material

Supplementary Figure 1 The co-expression network with thresholding power.

Supplementary Figure 2 PCR test of CRISPR/Cas9 vectors.

Supplementary Figure 3 The creation process of gene editing materials. **(A)** Seedlings of JIN668 sterilization. **(B)** Co-culture stage. **(C)** Selective culture medium. **(D)** Callus generation. **(E)** Differentiation culture stage. **(F)** Embryological callus. **(G)** Rooting culture stage. **(H)** Hydroponic seedling. **(I)** T0 generation regeneration seedlings grown in the greenhouse.

Supplementary Table 4 The differentially expressed genes between wild-type and lncD09 mutant plants.

Supplementary Table 5 The differentially expressed genes between wild-type and lncA07 mutant plants.
