# Supplementary figures and images for: Identification and Functional Analysis of lncRNA by CRISPR/Cas9 During the Cotton Response to Sap-Sucking Insect Infestation
Source: Front Plant Sci. 2022 Feb 23;13:784511. doi: 10.3389/fpls.2022.784511 (PMC8905227; doi:10.3389/fpls.2022.784511)

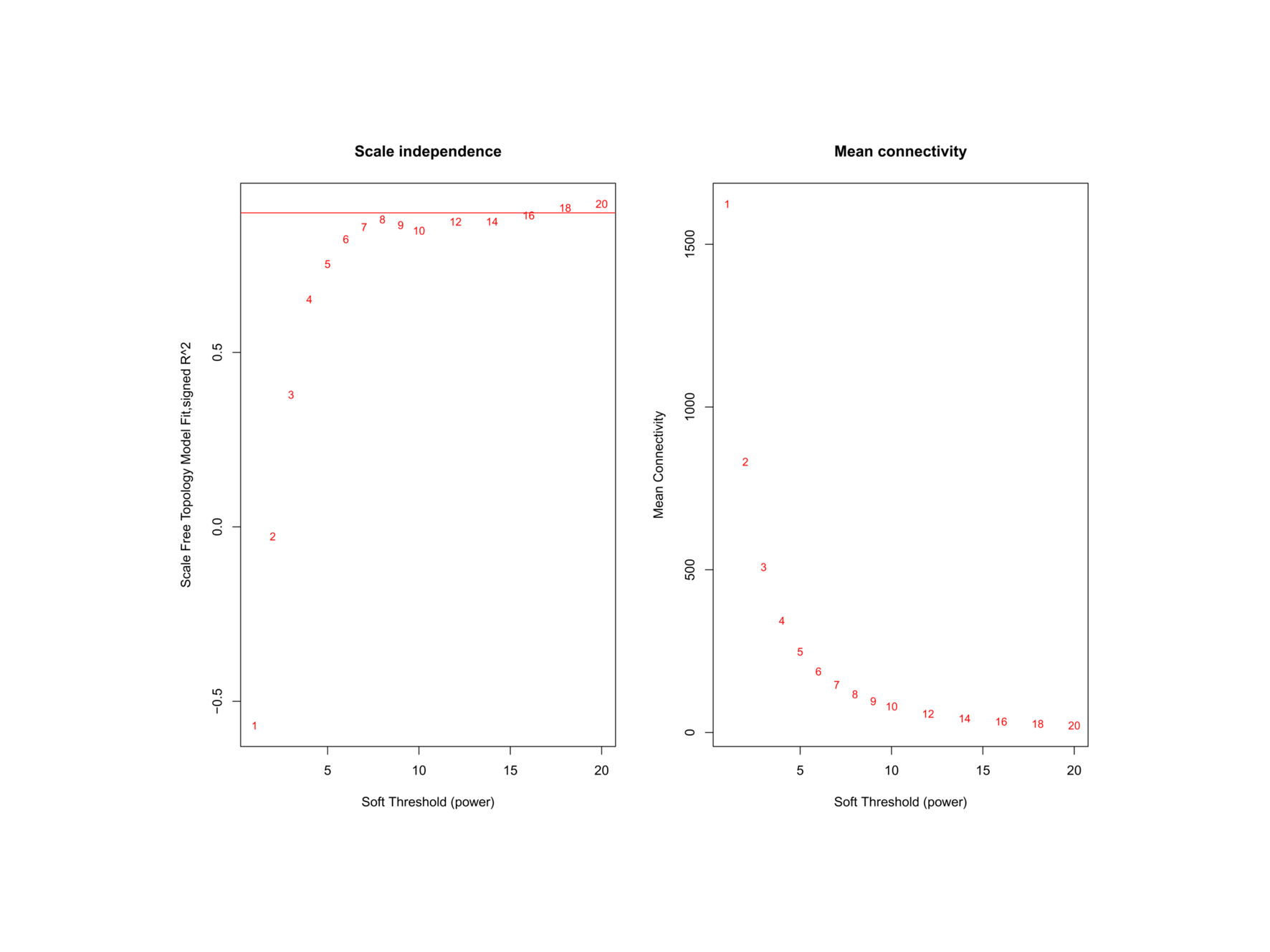

Supplement: Supplementary Figure 1 — The co-expression network with thresholding power. [file Image_1.TIFF]

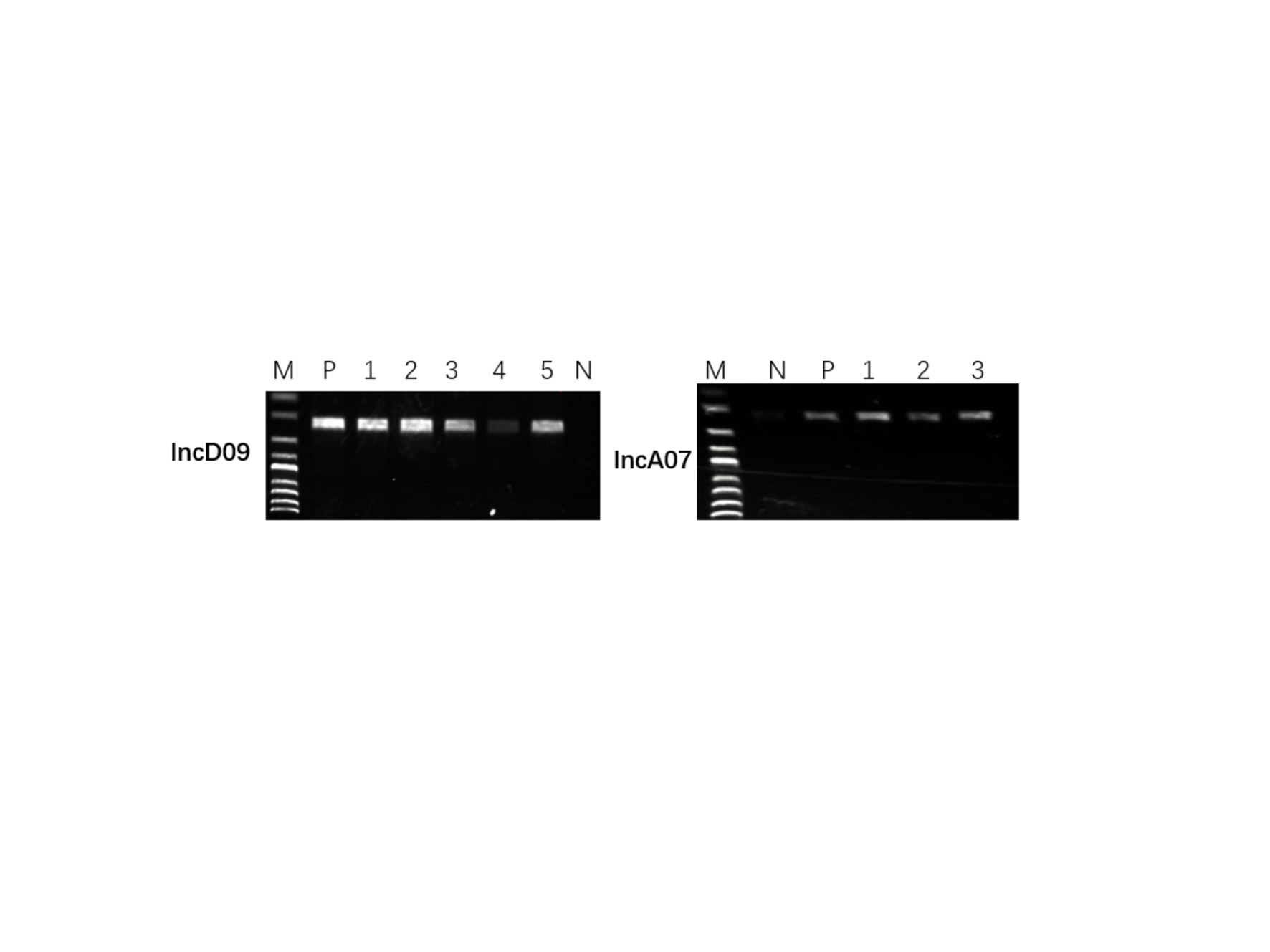

Supplement: Supplementary Figure 2 — PCR test of CRISPR/Cas9 vectors. [file Image_2.TIFF]

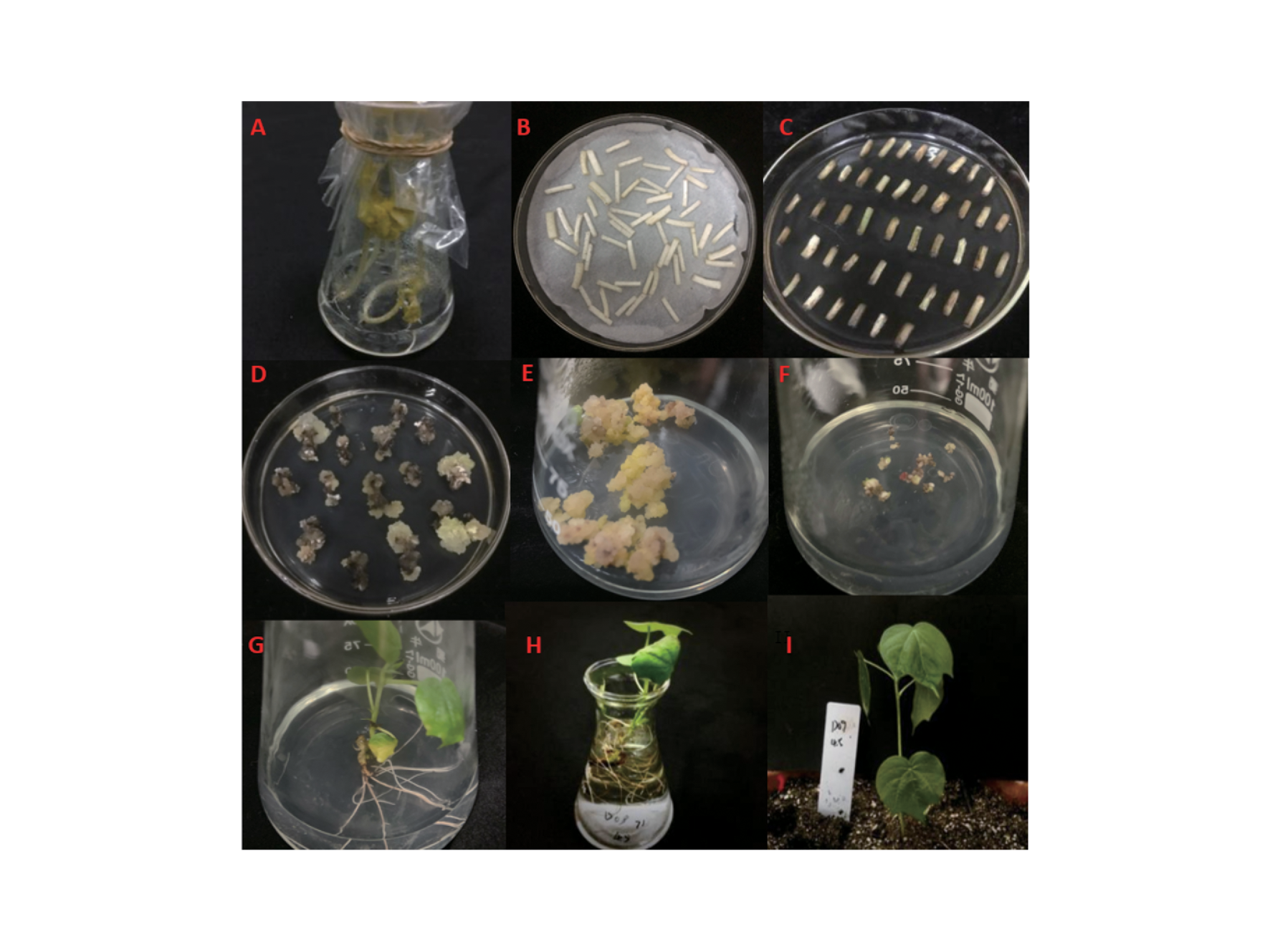

Supplement: Supplementary Figure 3 — The creation process of gene editing materials. (A) Seedlings of JIN668 sterilization. (B) Co-culture stage. (C) Selective culture medium. (D) Callus generation. (E) Differentiation culture stage. (F) Embryological callus. (G) Rooting culture stage. (H) Hydroponic seedling. (I) T0 generation regeneration seedlings grown in the greenhouse. [file Image_3.TIF]
