# Supplementary material for: Identification and Functional Analysis of lncRNA by CRISPR/Cas9 During the Cotton Response to Sap-Sucking Insect Infestation
Source: Front Plant Sci. 2022 Feb 23;13:784511. doi: 10.3389/fpls.2022.784511 (PMC8905227; doi:10.3389/fpls.2022.784511)
Supplement: Supplementary file 6 [file Table_1.DOCX]

Table S1 Primers used for lncD09 vectors construction

| Purpose | Sequence |
| --- | --- |
| Frist time PCR | pRGEB32-7 F:AAGCATCAGATGggcaAACAAAGCACCAGTGGTCTAG |
|  | R:ACTGTTCCTTGCTACATATGtgcaccagccgggaat  2F:CATATGTAGCAAGGAACAGTgttttagagctagaaata  2R:TGTCCTTGAGCTGTGATACCtgcaccagccgggaat |
| Second time PCR | inf pRGEB32-7  F: AAGCATCAGATGGGCAAACAAA |
|  | R:ttctagctctaaaacTGTCCTTGAGCTGTGATACC |
