# Supplementary material for: Identification and Functional Analysis of lncRNA by CRISPR/Cas9 During the Cotton Response to Sap-Sucking Insect Infestation
Source: Front Plant Sci. 2022 Feb 23;13:784511. doi: 10.3389/fpls.2022.784511 (PMC8905227; doi:10.3389/fpls.2022.784511)
Supplement: Supplementary file 8 [file Table_3.DOCX]

Table S3 Primers used for Hi-Tom method

lncD09-sgRNA1:

F:ggagtgagtacggtgtgcCAATCTTACGGAAAAAAACTACCC

R: gagttggatgctggatgg TGGCTTATACCAAGTAGGAAACAA

lncD09-sgRNA2:

F:ggagtgagtacggtgtgcACAAAAAATCACGACCTCCCTC

R: gagttggatgctggatggGCGTCGCTACCTTCTGCACT

lncA07-sgRNA1:

F:ggagtgagtacggtgtgcATAAGGAATGCCTCAATTGTAAAC

R: gagttggatgctggatggGCGGCTCTGGTTTTAGATGTA

lncA07-sgRNA2:

F:ggagtgagtacggtgtgcGTCATCTCGTTTGATGTTTCTTAC

R: gagttggatgctggatggTACCAGAAACTTCAATCTCCCT
